# Mutations in genes encoding complement inhibitors *CD46 *and *CFH *affect the age at nephritis onset in patients with systemic lupus erythematosus

**DOI:** 10.1186/ar3539

**Published:** 2011-12-15

**Authors:** Andreas Jönsen, Sara C Nilsson, Emma Ahlqvist, Elisabet Svenungsson, Iva Gunnarsson, Karin G Eriksson, Anders Bengtsson, Agneta Zickert, Maija-Leena Eloranta, Lennart Truedsson, Lars Rönnblom, Gunnel Nordmark, Gunnar Sturfelt, Anna M Blom

**Affiliations:** 1 Section of Rheumatology, Department of Clinical Sciences Lund, Lund University, Kioskgatan 3, SE-221 85 Lund, Sweden; 2 Section of Medical Protein Chemistry, Department of Laboratory Medicine Malmö, Lund University, S-205 02 Malmö, Sweden; 3 Section of Diabetes and Endocrinology, Department of Clinical Sciences Malmö, Lund University, S-205 02 Malmö, Sweden; 4 Rheumatology Unit, Department of Medicine, Karolinska University Hospital, Karolinska Institute, S-171 76 Stockholm, Sweden; 5 Section of Rheumatology, Department of Medical Sciences, Uppsala University, SE-751 Uppsala, Sweden; 6 Department of Laboratory Medicine, Section of Microbiology, Immunology and Glycobiology, Lund University, Sölvegatan 23, 223 62 Lund, Sweden

## Abstract

**Introduction:**

Inherited deficiencies of several complement components strongly predispose to systemic lupus erythematosus (SLE) while deficiencies of complement inhibitors are found in kidney diseases such as atypical hemolytic uremic syndrome (aHUS).

**Methods:**

The exons of complement inhibitor genes *CD46 *and *CFH *(factor H) were fully sequenced using the Sanger method in SLE patients with nephritis originating from two cohorts from southern and mid Sweden (*n *= 196). All identified mutations and polymorphisms were then analyzed in SLE patients without nephritis (*n *= 326) and in healthy controls (*n *= 523).

**Results:**

We found nonsynonymous, heterozygous mutations in *CFH *in 6.1% patients with nephritis, in comparison with 4.0% and 5.4% in patients without nephritis and controls, respectively. No associations of SLE or nephritis with common variants in *CFH *(V62I/Y402H/E936D) were found. Furthermore, we found two nonsynonymous heterozygous mutations in *CD46 *in SLE patients but not in controls. The A353V polymorphism, known to affect function of *CD46*, was found in 6.6% of nephritis patients versus 4.9% and 6.1% of the non-nephritis SLE patients and controls. The presence of mutations in *CD46 *and *CFH *did not predispose to SLE or nephritis but was associated with earlier onset of nephritis. Furthermore, we found weak indications that there is one protective and one risk haplotype predisposing to nephritis composed of several polymorphisms in noncoding regions of *CD46*, which were previously implicated in aHUS.

**Conclusions:**

SLE nephritis is not associated with frequent mutations in *CFH *and *CD46 *as found in aHUS but these may be modifying factors causing earlier onset of nephritis.

## Introduction

Systemic lupus erythematosus (SLE) is a complex and heterogeneous autoimmune disease affecting multiple organs that is characterized by circulating antibodies to nuclear antigens. Many studies have demonstrated a strong genetic component to SLE. Several susceptibility loci have recently been identified in genes encoding proteins involved in many immunological pathways [1], including B-cell signaling and development, cytokine production [2], the type I interferon pathway [3,4], signaling through Toll-like receptors, and neutrophil function [5].

One of the immune system cascades involved in the etiopathogenesis of SLE is the complement system. Complement is a pivotal part of the innate immunity, protecting the host from infections and participating in many processes that maintain tissue homeostasis [6]. In active SLE, immune complex deposition and complement activation contribute to tissue inflammation and damage. On the other hand, inherited deficiencies of complement components such as C1, C2 and C4 strongly predispose to the development of SLE [7]. This predisposition may be because an intact complement system is important for opsonization and clearance of apoptotic and necrotic cells as well as immune complexes, and thus is important for the prevention of autoimmunity. Additionally, complement is involved in B-cell maturation, differentiation and tolerance. Complement is also involved in microbial defense and thus may be related to SLE exacerbations caused by infections.

Complement is a proteolytic cascade that must be tightly regulated by several soluble and membrane-bound inhibitors in order to prevent damage to own tissues. These inhibitors are typically built of complement control protein (CCP) domains and are mainly encoded by the RCA (regulators of complement activation) gene cluster located on the long arm of chromosome 1. The present study was focused on the genes encoding two such proteins: *CD46 *encoding membrane cofactor protein (MCP), and *CFH *encoding factor H (FH). MCP is a cell-bound inhibitor, while FH circulates in blood. Nearly all human cell types, with the exception of erythrocytes, express MCP. This protein acts as a cofactor to serine proteinase factor I (FI), which is able to degrade activated complement components C3b and C4b and thereby to inhibit all pathways of complement. MCP is composed of four CCP domains followed by a serine/threonine-rich region, a transmembrane domain and a small intracellular domain. FH is the major soluble inhibitor of the alternative pathway of complement, serving as a cofactor to FI in degradation of C3b. FH is composed of 20 CCP domains, some of which have a high degree of homology with FH-related proteins 1 to 5 (CFHR1 to CFHR5).

Immune complexes generated in SLE can be passively trapped in kidney glomeruli but also directly bound to glomerular structures, causing a wide range of renal lesions including glomerulonephritis, vasculopathy and tubulointerstitial disease [8]. Defects in adequate inhibition of complement caused by inherited or acquired deficiencies of complement inhibitors could thus be involved in development and exacerbations of SLE nephritis. Importantly, inherited defects in complement inhibitors have already been associated with several kidney diseases. Complete deficiency of FH leads to membranoproliferative glomerulonephritis [9], complete deficiency of FI results in glomerulonephritis [10], while heterozygous mutations in genes encoding FH, FI and MCP result in atypical hemolytic uremic syndrome (aHUS).

Because of the well-established role of complement in SLE and the frequent genetic deficiencies of complement inhibitors in kidney diseases, we hypothesized that mutations or polymorphisms in complement inhibitors may be associated with SLE, and in particular with SLE nephritis. Recent genome-wide association studies have been successful in identifying a number of SLE-associated genes [1,11-13]. However, these studies only investigated the effect of single common variants. In the present study we performed Sanger sequencing of all exons in the *CD46 *and *CFH *genes in two cohorts of SLE patients collected in southern and mid Sweden to identify rare variants with a potential effect on SLE and SLE nephritis. We also analyzed the effect of haplotypes in *CFH *and *CD46 *that are known to affect related traits.

## Materials and methods

### Patients and controls

We studied two cohorts of unrelated SLE patients originating from southern Sweden (Lund) and mid Sweden (Uppsala and Stockholm). The Lund cohort consisted of 164 unselected consecutive SLE patients (21 men/143 women) treated at Skåne University Hospital in Lund and who agreed to participate in the study. The Uppsala cohort consisted of 35 SLE patients (four men/31 women) with nephritis and 35 age-matched SLE patients without nephritis (four men/31 women) all treated at the University Hospital in Uppsala. The Stockholm cohort of 288 patients (30 men/258 women) included unselected consecutive SLE patients treated at Karolinska University Hospital in Stockholm. Because of the very close geographical vicinity of Uppsala and Stockholm these two cohorts were pooled into one cohort (mid Sweden) composed finally of 358 SLE patients (38 men/320 women).

All SLE patients included in the study fulfilled four or more of the American College of Rheumatology classification criteria for SLE [14]. The majority (90.5%) of patients were Caucasians. The clinical characteristics of the patients are presented in Table 1. Additionally, 186 (37 men/149 women; southern Sweden) and 70 (8 men/62 women; Uppsala) healthy blood donors matched for sex and age were enrolled as controls. Controls (*n *= 267) originating from Stockholm (20 men/247 women) were individually sex-matched and age-matched population controls.

**Table 1:** Patient characteristics

	Southern Sweden	Mid Sweden
Number of patients	164	358
Women	143 (87)	320 (89)
Age at diagnosis (years)	46.0 ± 13.2	31.8 ± 13.6
Age at nephritis onset (years)	33.8 ± 14.6	33.6 ± 14.9
SLE duration (years)	16.0 ± 9.7	17.7 ± 11.0
Number of SLE criteria	5.9 (4 to 10)	5.8 (4 to 10)
Malar rash	95 (58)	190 (53)
Discoid rash	61 (37)	59 (16)
Photosensitivity	116 (71)	195 (54)
Oral ulcer	42 (26)	114 (32)
Arthritis	132 (80)	299 (84)
Serositis	88 (54)	158 (44)
Nephritis	43 (26)	153 (43)
Neurological disorder	15 (9)	45 (13)
Hematological disorder	87 (53)	241 (61)
Immunological disorder	121 (74)	265 (74)
Antinuclear antibodies	164 (100)	356 (99)

Data presented as *n *(%), mean ± standard deviation or mean (range). SLE, systemic lupus erythematosus.

The local ethic committees of Lund University and Uppsala University and the regional ethical review board in Stockholm approved the study, and subjects' consent was obtained according to the Declaration of Helsinki.

### Genotyping

DNA sequencing using the Sanger dideoxy method was performed by Polymorphic DNA Technologies (Alameda, CA, USA). All exons including at least 20 flanking intron nucleotides were analyzed for *CFH *and *CD46 *in patients with nephritis (southern Sweden, *n *= 43; mid Sweden, *n *= 153). Thereafter, all exons containing mutations and polymorphisms identified in *CFH *and *CD46 *in the previous step were also sequenced in SLE patients without kidney involvement (southern Sweden, *n *= 121; mid Sweden, *n *= 205) as well as controls (southern Sweden, *n *= 186; mid Sweden, *n *= 337). Finally, some synonymous SNPs in *CD46 *that were previously associated with aHUS (rs2796267, rs2796268, rs1962149, rs859705 and rs7144) and form the *CD46_ggaac _*haplotype were genotyped in all SLE patients and controls. The mean base call rate for the project was 99%. All identified SNPs were in Hardy-Weinberg equilibrium (*P *> 0.01). The protein numbering system used includes signal sequences (that is, the first methionine numbered as amino acid 1).

### Statistical analysis

Statistical significances of differences in mutation frequencies (listed in Table 2) between SLE patients and matched controls, between SLE patients with nephritis and controls, as well as between SLE patients with nephritis and SLE patients without nephritis were calculated using Fischer's exact test. The association of the mutations in *CFH *and *CD46 *with the year of onset of nephritis was analyzed using the nonparametric Mann-Whitney test with a two-tailed *P *value. The calculations reported included all patients irrespective of their ethnic background, but similar values were obtained when SLE patients with nephritis of non-Caucasian origin were excluded (in total, three nephritis patients carrying mutations and 19 patients without). We also estimated the effect of selected SNPs (frequency higher than 5% in the studied population) by multiple logistic regression analysis corrected for sex and center. SNP haplotypes (frequency higher than 1% in the studied population) were analyzed by a haplotype-based multiple logistic regression test corrected for sex and center. All statistical analyses were performed using PLINK version 1.06 [15]. Max T permutation (10,000 permutations) was used to determine exact single SNP and SNP haplotype *P *values. A two-tailed *P *value of 0.05 was considered significant. Only Caucasian patients were included in the SNP and haplotype analyses (four SLE patients and 46 SLE patients were excluded from the southern and mid Sweden cohorts, respectively). No adjustments for multiple comparisons were conducted due to the exploratory nature of this study in which we tested a limited number of comparisons based on *a priori *hypotheses.

**Table 2:** Nonsynonymous mutations found in *CD46 *and *CFH *genes

Gene	SNP number	**Amino acid**^**a**^	cDNA ATG + 1	Genomic DNA	Domain	Frequency in SLE patients with/without nephritis (%(*n*))		Frequency in controls (%(*n*))		Disease association/function	References
								
						Southern Sweden (*n *= 43)/(*n *= 121)	Mid Sweden (*n *= 153)/(*n *= 205)	Southern Sweden (*n *= 186)	Mid Sweden (*n *= 337)		
*CD46*	Novel	S13F	c.38C > T	Exon 1	Signal peptide	0(0)/0(0)	0.6(1)/1.0(2)	0(0)	0(0)	Healthy controls	[16]
*CD46*	Novel	A219V	c.656C > T	Exon 5	CCP3	2.3(1)/0(0)	0(0)/0(0)	0(0)	0(0)	n.d.	
*CD46*	rs35366573	A353V	c.1058 C > T	Exon 11	Trans-membrane	11.6(5)/5.0(6)	5.2(8)/3.0(10)	4.8(9)	6.8(23)	aHUS, HELLP, GN with C3 deposits and MPGN	[27,39-41]
*CFH*	Novel	N29D	c.85A > G	Exon 2	CCP1	0(0)/0(0)	0(0)/0(0)	0(0)	0.3(1)	n.d.	
*CFH*	Novel	Q400K	c.1198C > A	Exon 9	CCP7	0(0)/0.8(1)	0(0)/0(0)	0.5(1)	0(0)	aHUS	[42]
*CFH*	Novel	N516K	c.1548T > A	Exon 11	CCP9	2.3(1)/0(0)	0(0)/0(0)	0(0)	0.3(1)	n.d.	
*CFH*	Novel	N556S	c.1667A > G	Exon 11	CCP9	0(0)/0.8(1)	0(0)/0(0)	0(0)	0(0)	n.d.	
*CFH*	Novel	Q950H	c.2850G > T	Exon 18	CCP16	4.7(2)/1.7(2)	0(0)/0.5(1)	1.6(3)	0.6(2)	aHUS	[43-45]
*CFH*	Novel	F960S	c.2879T > A	Exon 18	CCP16	0(0)/0(0)	0.7(1)/0(0)	0(0)	0(0)	n.d.	
*CFH*	rs35274867	N1050Y	c.3148A > T	Exon 20	CCP18	2.3(1)/2.5(3)	3.9(6)/2.6(4)	2.2(4)	4.7(16)	aHUS, AMD, MPGN2	[44,46-48]
*CFH*	rs62625015	Q1076E	c.3226C > G	Exon 20	CCP18	0(0)/0.8(1)	0.7(1)/0(0)	0(0)	0(0)	aHUS	[44,48]

All mutations were found in a heterozygous form. The difference in mutation frequencies calculated for systemic lupus erythematosus (SLE) patients versus controls and for SLE patients with nephritis versus controls or versus SLE patients without nephritis were not statistically significant according to Fischer's exact text when calculated for all study participants together or divided into the thee cohorts. aHUS, atypical hemolytic uremic syndrome; AMD, age-related macular degeneration; CCP, complement control protein; HELLP, hemolysis, elevated liver enzymes, and low platelets; n.d., not described; GN, glomerulonephritis; MPGN, membranoproliferative glomerulonephritis; MPGN2, membranoproliferative glomerulonephritis type 2. ^a^Numbering including signal sequence.

## Results

### Mutations and polymorphisms identified in the *CD46 *gene

Analysis of all *CD46 *exons in 196 patients with nephritis (southern Sweden, *n *= 43; mid Sweden, *n *= 153) revealed three nonsynonymous mutations in the coding exons. One of these mutations (S13F), found in heterozygous form in three SLE patients, was localized in the signal peptide and previously found in healthy controls [16] even though the latter was not the case in the current study. The other mutation (A219V), found in one patient, was localized in the CCP3 domain. Furthermore, we found that 11.6% and 5.2% of the patients with nephritis from the southern and mid Sweden cohorts carried the A353V polymorphism. In the patients without nephritis, only 5.0% from southern Sweden and 3.0% from mid Sweden carried the polymorphism; whereas in the control groups, 4.8% and 6.8% of individuals had the polymorphism. The differences in the A353V polymorphism frequencies between SLE patients and controls or SLE patients with nephritis and those without nephritis or healthy controls were not statistically significant according to Fisher's exact test (Table 2).

Since several studies identified a specific SNP haplotype block spanning *CD46 *(*CD46_ggaac_*), which is overrepresented in aHUS patients [17,18], we assessed these SNPs (rs2796267/-652 A > G, rs2796268/-366 A > G, rs1962149/IVS9-78 G > A, rs859705/IVS12+638 G > A, rs7144/c.4070 T > C) in our cohorts. Only haplotypes with frequencies of at least 1% in our cohorts were analyzed. We found weak indications that the aHUS-associated *CD46_ggaac _*haplotype could perhaps be protective for SLE, and in particular for SLE with nephritis, while the *CD46_agaac _*could represent risk haplotype. The obtained *P *values, however, were borderline significant (Table 3).

**Table 3:** Associations of haplotypes in *CD46 *with SLE and SLE nephritis

		Frequency				SLE nephritis versus healthy controls		SLE versus healthy controls	
		
Cohort	*CD46 *haplotype	All SLE patients	SLE with nephritis	SLE without nephritis	Healthy controls	Odds ratio	*P *value	Odds ratio	*P *value
Southern Sweden	GGAAC	0.3068	0.2457	0.3271	0.3512	0.60	0.076	0.81	0.22
Southern Sweden	AGAAC	0.0901	0.0418	0.1062	0.0573	0.66	0.52	1.53	0.17
Southern Sweden	GAGGT	0.0901	0.0918	0.0895	0.0627	1.59	0.34	1.41	0.26
Southern Sweden	AAGGT	0.4974	0.5707	0.473	0.5099	1.3	0.31	0.99	0.93
Mid Sweden	GGAAC	0.3242	0.3251	0.3238	0.37	0.81	0.18	0.82	0.090
Mid Sweden	AGAAC	0.0892	0.112	0.0723	0.0643	1.93	0.011	1.45	0.093
Mid Sweden	GAGGT	0.0636	0.0555	0.0695	0.0647	0.84	0.60	0.96	0.84
Mid Sweden	AAGGT	0.5117	0.5005	0.5204	0.4875	1.07	0.68	1.12	0.34
Combined cohort	GGAAC	0.3184	0.3063	0.3255	0.3633	0.76	0.047	0.82	0.044
Combined cohort	AGAAC	0.0895	0.096	0.0856	0.0618	1.59	0.056	1.48	0.030
Combined cohort	GAGGT	0.0725	0.0644	0.0772	0.0641	1	1	1.09	0.64
Combined cohort	AAGGT	0.5069	0.5161	0.5017	0.4954	1.12	0.38	1.07	0.48

SLE, systemic lupus erythematosus.

### Mutations and polymorphisms identified in the *CFH *gene

Analysis of all *CFH *exons in the SLE patients identified seven nonsynonymous mutations. In addition, a novel nonsynonymous mutation (N29D) was found in a healthy individual. All of the mutations occurred in heterozygous state. The Q400K, Q950H, N1050Y and Q1076E mutations were previously described in patients with aHUS while the N29D, N516K and N556S mutations appear to have never been reported. In the current study these three mutations were also found in healthy individuals at similar frequencies to those in SLE patients. Analysis of three common coding SNPs in *CFH *(V62I/c.184G > A, Y402H/c.1204T > C, E936D/c.2808G > T) showed no significant association with SLE or SLE nephritis. Furthermore, no associations of the haplotypes formed by these SNPs and SLE/SLE nephritis were found.

### Mutations in *CD46 *and *CFH *influence age at onset of nephritis

**Figure 1:**
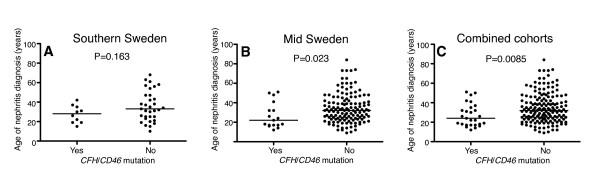
**Age at onset of nephritis in systemic lupus erythematosus patients**. Median (range) age of nephritis diagnosis for systemic lupus erythematosus patients with or without *FH/CD46 *mutations, respectively: **(A) **Southern Sweden cohort, 28 (15 to 42) years and 33 (10 to 68) years; **(B) **mid Sweden cohort, 22 (12 to 51) years and 32 (9 to 84) years; and **(C) **combined cohort, 24 (12 to 51) years and 32 (9 to 84) years. Horizontal line, median for each dataset. Statistical significances of differences between patients carrying mutations and those without *CD46 *and *CFH *were evaluated by nonparametric Mann-Whitney test with a two-tailed *P *value.

Mutations found in *CD46 *and *CFH *(listed in Table 2) were found to affect the age at onset of nephritis in the analyzed SLE patients (Figure 1). The effect was statistically significant in the larger cohort from mid Sweden (*P *= 0.023) and the significance increased when the two cohorts were analyzed simultaneously (*P *= 0.0085). The median age of nephritis onset in the SLE patients carrying *CD46 *and *CFH *mutations in the combined cohorts was 24 years (range 12 to 51) compared with 32 years (range 9 to 84) in the patients free of the mutations. Thus, even though these mutations do not appear to be causative factor for nephritis, they may contribute to the earlier onset of the disease. The multiple regression analysis did not show significant association between onset of nephritis and *CD46 *(rs2796267/-652 A > G, rs2796268/-366 A > G, rs1962149/IVS9-78 G > A, rs859705/IVS12+638 G > A, rs7144/c.4070 T > C) or *CFH *(V62I/c.184G > A, Y402H/c.1204T > C, E936D/c.2808G > T) haplotypes.

## Discussion

Because complement is intricately involved in SLE and mutations/polymorphisms in complement inhibitors are associated with a number of kidney diseases, we hypothesized that SLE patients, particularly those with nephritis, may also carry such genetic defects. Kidney involvement is common in SLE, occurring in up to 25 to 30% of affected Caucasian adults at some stage during the course of their disease [19]. In most cases, renal disease develops within the first 3 years following diagnosis. Lupus nephritis still remains a main morbidity and mortality determinant for patients with SLE and the current treatment is not satisfactory considering the rate of success and adverse side effects. If inadequate control of the complement system caused by genetic predisposition is involved in SLE nephritis, it could become an additional indication for use of the emerging complement inhibitors such as eculizumab, which is a monoclonal antibody inhibiting cleavage and activation of C5.

To test our hypothesis we sequenced all exons of the *CD46 *and *CFH *genes in two Swedish cohorts of SLE patients. Importantly, we could relate the obtained genetic data to a carefully characterized clinical history of the patients. Interestingly, even though we did not find significant association between the presence of mutations in *CD46 *and *CFH *and SLE or SLE nephritis, we observed that these mutations are associated with a younger age at onset of glomerulonephritis. The treatment of all patients with SLE was determined by the clinical manifestations and was similar according to clinical praxis in the rheumatology clinics in southern and mid Sweden, in accordance with EULAR recommendations [20]. There is therefore no reason to believe that different treatment regimens could have affected the time of nephritis onset.

The observed earlier onset of nephritis in SLE patients carrying mutations in *CD46 *and *CFH *is consistent with a recently published study showing that FH deficiency accelerates development of lupus nephritis in MRL-lpr mice, which share many features of human SLE including production of autoantibodies and consumptive hypocomplementemia [21]. Histopathologic findings in these animals included marked deposition of immune complexes containing C3 in glomerular subendothelial, mesangial and subepithelial locations and glomerular inflammation with infiltrated neutrophils and macrophages [21]. Complement has complicated and paradoxical roles in SLE. Although deficiencies of early components of the complement cascade are associated with development of SLE due to their role in clearance of dying cells and tissue debris [7] and their importance in development of tolerance [22], clearance of immune complexes [23] and cytokine regulation [24], activation of complement at later stages of C3 and beyond also contributes to the pathogenesis of SLE. Studies using experimental models showed that development of lupus nephritis was dependent on generation of C5a in glomeruli [25] and on the presence of iC3b in glomerular immune complexes [26]. Such observed complement activation in glomerulus was triggered by immune complexes. MCP and FH are both localized to glomerular capillary walls, where they attenuate complement activation under normal conditions. However, this level of protection may be compromised by inherited or acquired deficiency of these proteins.

The A353V polymorphism in MCP has previously been shown to affect the ability of MCP to control the alternative pathway activation [27]. This conservative amino acid substitution in the transmembrane domain did not affect the ability of recombinant MCP to bind C3b/C4b and to act as a cofactor to FI in the fluid phase. The mutant was defective in its regulatory activity, however, when embedded in the membrane - although the mechanism underlying this impairment of function was not defined. The A353V polymorphism has been identified in several patients with renal pathology such as aHUS, glomerulonephritis with C3 deposits and HELLP syndrome (hemolysis, elevated liver enzymes, and low platelets) [27]. We did not detect association of this polymorphism with SLE in general or SLE nephritis in the current study, however, and we found the polymorphism in 5.8% healthy controls analyzed in the present study and in 3.1% of 192 healthy controls of Caucasian origin included in the study of spontaneous pregnancy loss (manuscript in preparation, Mohlin F, Mercier E, Fremeaux-Bacchi V, Liszewski K, Atkinson JP, Gris JC, Blom AM). The NCBI database estimates 2% frequency for this SNP in European populations [28]. Taken together, these frequencies suggests that the A353V polymorphism in MCP cannot be a strong causative factor for SLE/SLE nephritis, and most probably not aHUS either, but could be a modifying factor in the presence of additional defects in complement regulation often observed in these patients.

The *CD46_ggaac _*haplotype has been previously shown to be overrepresented in aHUS patients compared with controls (odds ratio = 2.68). Interestingly, this association was mainly due to the aHUS patients with mutations in *CFH*, *CD46 *or *CFI *(odds ratio = 5.25), whereas aHUS patients without identified mutations in *CFH*, *CD46 *or *CFI *showed no differences with the control group [17]. The functional consequences of the five synonymous SNPs in the *CD46_ggaac _*haplotype have not been fully elucidated, but it has been shown that the rs2796267 and the rs2796268 SNPs located in the promoter region are involved in the transcriptional activity potentially due to disruption of the CBF-1/RBP-Jk binding site [17]. Two other SNPs in the *CD46_ggaac _*haplotype block are intronic, while the third lies in the 3' UTR. In the current study we found weak indication that the *CD46_ggaac _*haplotype could perhaps be protective for SLE, and in particular SLE with nephritis, whereas the *CD46_agaac _*could represent a risk haplotype. These indications must now be assessed in a larger patient material before final conclusions are made.

Several nonsynonymous polymorphisms in *CFH *have been linked to age-related macular degeneration [29,30] and susceptibility to meningococcal disease [31], but not to rheumatoid arthritis [32] or coronary heart disease [33]. A large number of studies have shown that I62V polymorphism in CCP1 of FH affects its FI cofactor activity and ability to accelerate decay of convertases [34,35] and that the Y402H polymorphism in CCP7 affects binding of FH to several ligands such as C-reactive protein, DNA, dying cells and heparin [36,37]. The effect of the D936E polymorphism in CCP16, however, has not yet been analyzed. In the current study we did not detect any significant associations with these three SNPs in *CFH *nor any of the haplotypes thereof. This finding is consistent with a recent publication assessing association of genetic variants in *CFH *with SLE susceptibility [38]. The authors studied 60 SNPs covering *CFH *and FH-related genes for association with SLE in over 15,000 case-control subjects from four ethnic groups. They found significant allelic associations with SLE in European Americans and African Americans, which could be attributed to an intronic *CFH *SNP and intergenic SNP between *CFHR1 *and *CFHR4 *rather than the exonic SNPs we studied.

## Conclusions

Taken together, SLE and SLE nephritis do not appear to be associated with frequent mutations in *CD46 *or *CFH *as reported for some other kidney diseases, but these mutations are likely to affect the year of onset of nephritis. Furthermore, the role of *CD46 *haplotypes still remains to be elucidated in a larger patient cohort.

## Abbreviations

aHUS: atypical hemolytic uremic syndrome; CCP: complement control protein; FH: factor H; FI: factor I; MCP: membrane cofactor protein; SLE: systemic lupus erythematosus; SNP: single nucleotide polymorphism; UTR: untranslated region.

## Competing interests

The authors declare that they have no competing interests.

## Authors' contributions

AMB initiated the collaborative project, designed the study, monitored data collection, designed the statistical analysis plan, and drafted and revised the paper. AJ collected clinical data and samples for patients in Lund, contributed to design of the study and revised the draft paper. SCN cleaned and assembled the data, performed statistical analyses, contributed to the design of the study and revised the draft paper. EA performed statistical analyses and revised the draft paper. ES collected clinical data and samples for patients at Karolinska Hospital, contributed to design of the study and revised the draft paper. IG collected clinical data and samples for patients at Karolinska Hospital, contributed to design of the study and revised the draft paper. KGE collected clinical data and samples for patients in Uppsala and revised the draft paper. AB collected clinical data and samples for patients in Lund and revised the draft paper. AZ collected clinical data and samples for patients at Karolinska Hospital and revised the draft paper. M-LE collected healthy control samples from Uppsala and revised the draft paper. LT provided samples of healthy controls from Lund and revised the draft paper. LR collected clinical data and samples for patients in Uppsala and revised the draft paper. GN collected clinical data and samples for patients in Uppsala, contributed to design of the study and revised the draft paper. GS collected clinical data and samples for patients in Lund, contributed to design of the study and revised the draft paper. All authors read and approved the final manuscript.
